# Supplementary material for: Functional Somatotopy of Lumbar Dorsal Rootlets and its Role in Selective Recruitment via Lateral Spinal Cord Stimulation
Source: bioRxiv. 2026 Jun 23:2026.06.18.733242. Preprint. [Version 1] doi: 10.64898/2026.06.18.733242 (PMC13320895; doi:10.64898/2026.06.18.733242)
Supplement: Supplement 1 [file NIHPP2026.06.18.733242v1-supplement-1.pdf]

bioRxiv preprint doi: https://doi.org/10.64898/2026.06.18.733242; this version posted June 23, 2026. The copyright holder for this preprint (which was not certified by peer review) is the author/funder, who has granted bioRxiv a license to display the preprint in perpetuity. It is made available under a CC-BY 4.0 International license.

SUPPLEMENTARY MATERIAL

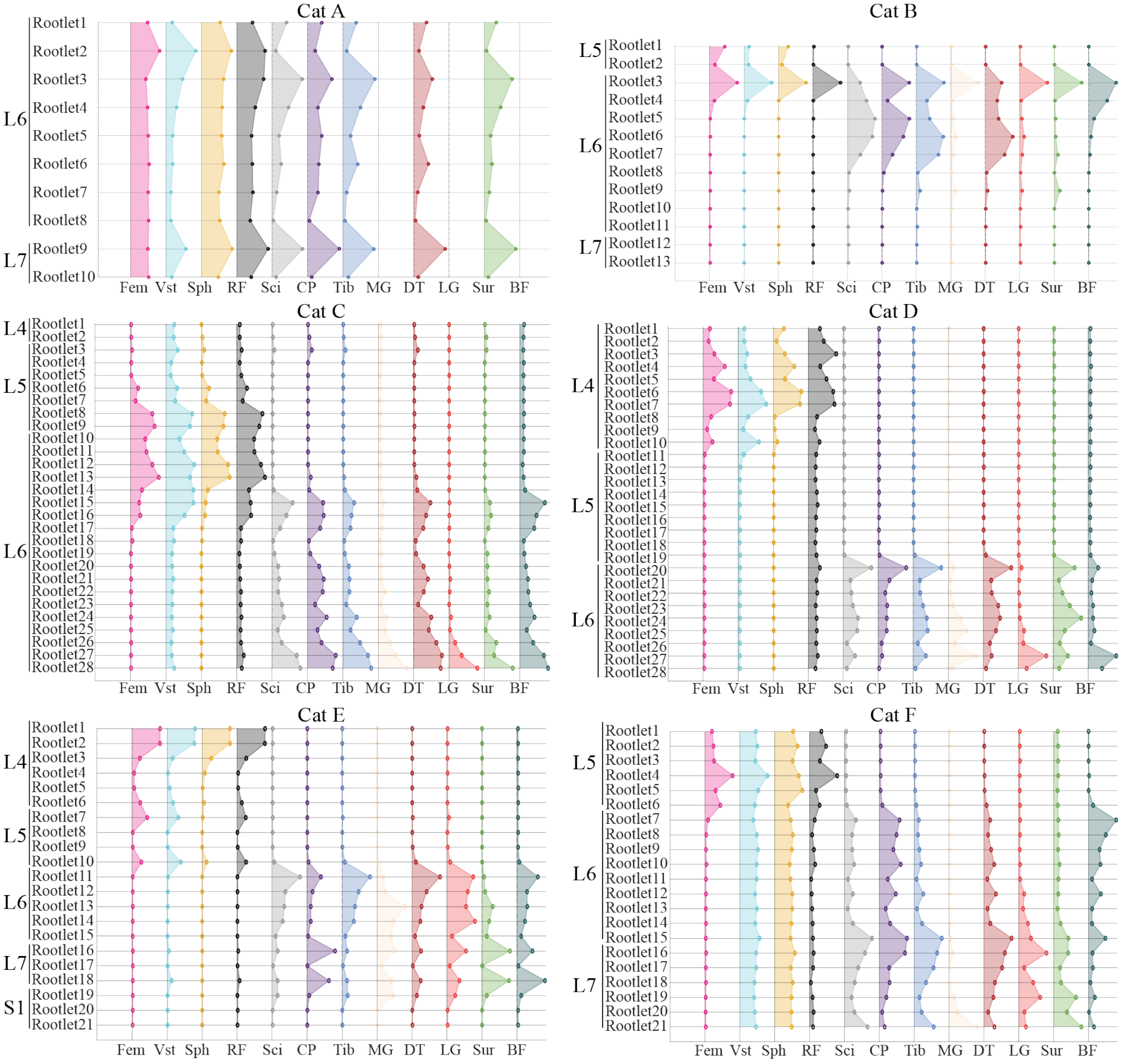

Supplementary Figure 1: Normalized nerve recruitment patterns across individual dorsal rootlets (DR) for all six cats. *Each plot shows the normalized compound action potential (CAP) amplitudes recorded from femoral (Fem), vasti (Vst), saphenous (Sph), rectus femoris (RF), sciatic (Sci), common peroneal (CP), tibial (Tib), medial gastrocnemius (MG), distal tibial (DT), lateral gastrocnemius (LG), sural (Sur), and biceps femoris (BF) nerves during DR stimulation at high stimulation amplitudes, comparable for each cat. Rootlets are arranged rostrocaudally within each spinal level (L4–S1). Broad and overlapping recruitment patterns within femoral and sciatic groups are evident across cats, demonstrating the lack of fine-grained somatotopic organization within functional groups.*
